# The complete genome of the bacterial isolate *Pseudomonas* sp. WMBT8 isolated from the freshwater fish “deepwater sculpin,” a glacial relic

**DOI:** 10.1128/mra.01395-25

**Published:** 2026-02-19

**Authors:** Frank E. Dailey, Andrew Kehm, Aiden Phalen, David A. Essig, Mark R. Vinson

**Affiliations:** 1 Department of Biology, University of Wisconsin-Sheboygan (now University of Wisconsin-Green Bay-Sheboygan Campus) 14763 , Sheboygan, Wisconsin, USA; 2 Department of Biology, Geneva College 35217, Beaver Falls, Pennsylvania, USA; 3 Retired Researcher 109724, Ashland, Wisconsin, USA; California State University San Marcos, San Marcos, California, USA

**Keywords:** *Pseudomonas*, deepwater sculpin, intestinal microbiota

## Abstract

We report here the genome sequence of the bacterium *Pseudomonas* sp. WMBT8 isolated from the gastrointestinal tract of the glacial relic freshwater fish *Myoxocephalus thompsonii* (deepwater sculpin). DNA sequencing results showed that strain WMBT8 had a single circular genome of 6.1 Mb with a GC content of 61%.

## ANNOUNCEMENT

Previously, we isolated omega-3 fatty acid producing *Shewanella* species from the gastrointestinal (GI) tracts of several cold freshwater fish (1). In search of novel producers, we investigated the GI tracts of deepwater sculpin since they exist in cold freshwater habitats and are likely glacial relics (2). The sculpin samples were caught in Lake Superior (Wisconsin, USA) at a depth of 60 m (map location 46.72 N, 91.87 W) with a water surface temperature of 10°C. GI tracts of the dead fish were isolated after ethanol was used to wash the skin and scalpel. Fish were dissected longitudinally, and GI tracts were removed sterilely and placed in trypticase soy broth then grown at 4°C. After 3 weeks, broth was diluted out on 50% marine agar (Difco Marine Broth 2216) plus 0.1% (wt/vol) tetrazolium chloride (Sigma-Aldrich) at 20°C. Such media often differentiate omega-3 fatty acid producers by their red color (3), although false positives can occur (1). Several red colonies were chosen for further study, and one, designated as strain WMBT8, was saved for further analysis, including genomic sequencing, since it showed antimicrobial properties (data not shown). DNA samples were prepared by swabbing a TSA plate streaked with strain WMBT8 and then extracting the DNA using PacBio’s Nanobind Tissue Kit. Genomic samples were fragmented (target size 15–18 kbp) using the Covaris g-TUBE following recommended PacBio procedures (4). Sequencing libraries were prepared following the PacBio SMRTbell Prep Kit 3.0 with the SMRTbell Barcoded Adapter Plate 3.0 (4). Pooled libraries underwent the PacBio binding and cleanup steps following the Sequel II Binding Kit 3.2 (5) and were loaded onto the PacBio Sequel IIe for a 15 h collection period, with options enabled to retain subreads and sequencing metrics. Demultiplexing, quality control, and adapter trimming were performed on instrument via Lima (v2.6) (6). Sequencing produced 116,066 reads yielding 1,127,439,573 bp with a median quality score of Q33. Default settings were used for all software.

The genome was assembled via HiFiASM (v0.16.1) (7). Bacterial assemblies underwent circularization via Circlator (v1.5.5), which set the first base in assembly using the *dnaA* gene (8). The genome was annotated via the NCBI Prokaryotic Genome Annotation Pipeline (v6.10 (9). Upon submission to NCBI, genome completeness and contamination were determined using CheckM (v1.2.3) (10) and taxonomy via average nucleotide identity (ANI) (11). Relevant assembly, annotation, and taxonomy statistics are provided in Table 1. Analysis via PHASTEST (v3.0) (12) revealed six prophages in the genome with five likely to be intact. Island Viewer (v4.0) (13) predicted that multiple genes were likely acquired via horizontal transfer, including adhesions, Type I secreted protein toxins, cold tolerance genes, a Type III and VI secretion system operon, and genes for phenazine synthesis. The genes for omega-3 fatty acid synthesis were absent.

**TABLE 1:** Genome assembly and taxonomy statistics for *Pseudomonas* species WMBT8

Genome attribute	*Pseudomonas* sp. WMBT8
Assembly length (Mb)	6.1
Number of contigs	1
Contig N50	1
Coverage (fold)	185
Circular assembly	Yes
G + C (%)	61
Total genes (#)	5,587
Protein coding genes (#)	5,432
Completeness (%)	100
Contamination (%)	0.08
Best genome match type strain, ANI	93.38% with *Pseudomonas shahriarae* strain SWR152 Genome accession number CP077085.1

We have isolated species of the *Pseudomonas* genus whose genome is highly related to *Pseudomonas shahriarae*’s genome, isolated from wheat. Both *P. shahriarae* and our isolate are members of the *P. gessardi* group (14) based on ANI (average nucleotide identity, Table 1).

## Data Availability

This Whole Genome Shotgun project has been deposited in DDBJ/ENA/GenBank under accession number CP188096. The version described in this paper is the first version. The SRA raw reads data are found at PRJNA1247135. The SRA is available under accession number SRR35898010. Data sets for predicted prophage insertions (PHASTEST v3.0) and genomic islands (Island Viewer v4.0) are deposited in Figshare (10.6084/m9.figshare.31032463).
